# Obfuscated Memory Malware Detection in Resource-Constrained IoT Devices for Smart City Applications

**DOI:** 10.3390/s23115348

**Published:** 2023-06-05

**Authors:** Sakib Shahriar Shafin, Gour Karmakar, Iven Mareels

**Affiliations:** 1 Centre for Smart Analytics (CSA), Federation University Australia, Ballarat, VIC 3350, Australia; gour.karmakar@federation.edu.au; 2 Institute of Innovation, Science and Sustainability (IISS), Federation University Australia, Ballarat, VIC 3350, Australia; i.mareels@federation.edu.au

**Keywords:** multiclass memory malware detection, deep learning, lightweight IoT security, embedded applications

## Abstract

Obfuscated Memory Malware (OMM) presents significant threats to interconnected systems, including smart city applications, for its ability to evade detection through concealment tactics. Existing OMM detection methods primarily focus on binary detection. Their multiclass versions consider a few families only and, thereby, fail to detect much existing and emerging malware. Moreover, their large memory size makes them unsuitable to be executed in resource-constrained embedded/IoT devices. To address this problem, in this paper, we propose a multiclass but lightweight malware detection method capable of identifying recent malware and is suitable to execute in embedded devices. For this, the method considers a hybrid model by combining the feature-learning capabilities of convolutional neural networks with the temporal modeling advantage of bidirectional long short-term memory. The proposed architecture exhibits compact size and fast processing speed, making it suitable for deployment in IoT devices that constitute the major components of smart city systems. Extensive experiments with the recent CIC-Malmem-2022 OMM dataset demonstrate that our method outperforms other machine learning-based models proposed in the literature in both detecting OMM and identifying specific attack types. Our proposed method thus offers a robust yet compact model executable in IoT devices for defending against obfuscated malware.

## 1. Introduction

The exponential increase in online activities, particularly during the Covid-19 pandemic [1], has led to significant growth toward building online infrastructures for numerous new and existing services, resulting in an unprecedented amount of data being processed and stored in cyberspace. For example, the global cloud services market is expected to grow from USD 396.1 billion in 2020 to USD 798.84 billion in 2025 at an annual growth rate of 14% [2]. As governments and organizations plan to deliver increasingly advanced services in smart city areas worldwide and factories embrace Industry 4.0 using Internet of Things (IoT) networks, it is projected that the number of IoT devices will surpass 30 billion by 2030. The multifold possibilities within a smart city concept include, among others, efficient and eco-friendly usage of technology to enhance the quality of services in healthcare [3], coordinated development boosted by smart economy [4], transportation, water, air quality management, waste management and surveillance. However, the main requirement of a smart city is connectivity across all devices and all aspects, which can only be a possibility if IoT is used on a mass scale [5], which is further proved by a recent study that shows about 70% of the USA businesses have invested heavily in Industrial IoT [6] and devices in smart homes and wearables, which directly contributes to the growth of smart cities.

To understand the vulnerabilities and underlying security challenges of smart city applications, it is crucial to understand the threats and vulnerabilities of the sensors on which those applications are built and associated threat mitigation strategies. Sensors detect and respond to physical stimuli, such as changes in temperature, pressure or motion, and convert them into analog or digital signals for subsequent processing and decision-making. From existing IoT-based smart city applications, it is evident that a wide range of sensors are now being used to operate and monitor the functions of smart cities that reach almost all facets of society. For example, one of the largest smart city projects was undertaken at the city of Santander, Spain, which utilizes Libelium’s Waspmote sensor platform [7], a versatile and modular sensor network analysis space, to monitor the environmental conditions of the city systems. In this project, Meshlium scanners are used as edge devices to gather data, while 750 sensors were deployed across 22 specified zones. Temperature, luminosity, carbon monoxide and sound noise sensors were employed. Libelium’s dedicated sensor nodes for smart cities, the Plug and Sense! Smart Cities PRO, equipped with two radios for 2.4 GHz communication and IEEE 802.15.4 protocol as standard are developed exclusively for this purpose. They contain BME280 temperature, humidity and pressure sensor, along with SCP v30 07 luminosity sensor, OPC-N3 dust sensor and CO-A4 carbon monoxide sensors [8]. Libelium’s next advanced project on smart city is currently under development at the city of Cartagena [9], where lampposts were integrated with air quality (OPC N3) and noise monitoring sensors equipped with fast cellular communication standards (5G). Utilizing OPC-N2 technology, AirSensa spearheads a large-scale air pollution monitoring endeavor in London, capturing precise measurements of PM1, PM2.5 and PM10 concentrations.

The Smart Nation Sensor Platform in Singapore [10] uses sensors to perform important tasks for smart city monitoring such as detecting water leaks, minimizing energy wastage and incident reporting on lightning strikes, where they integrated Vaisala GLD360 sensor network [11]. To monitor and keep track of environmental pollution, a major task for smart cities, Lecce, Italy is using air quality sensors in its “Integrated Energy Plan” to reduce CO2 emissions. A project on sustainable environment is taking place at Tartu, Estonia and Sonderberg, Norway who are utilizing smart meters and sensor-based monitoring [12] to improve energy efficiency in community housing and optimize solar energy usage. In Vitoria-Gasteiz, low-level sensors measuring temperature, humidity and CO2 are installed in dwellings, along with energy consumption measuring devices to improve living conditions [13]. The integration of sensors in smart city projects is essential for effective data-driven decision-making, and addressing the diverse challenges facing modern cities.

The exponential use of these sensors and IoT devices in smart cities and regular IoT environments as a whole present a unique set of challenges. Because of their resource-constrained nature and criticality of some applications, these devices are particularly vulnerable to complex and hard-to-detect attacks [14]. As most devices used in the lower tiers of IoT systems are left with up to 1 MB only in memory after installation of the operating system [15], resource-intensive security mechanisms are practically unfeasible. The importance of sectors where IoT devices are deployed, such as medical, energy and military operations, compounded by the scarcity of resources, makes them an easy target for cyber-attacks [16].

In the cyber attack landscape, malware is the most vicious threat to security. Their ability to stay undetected in systems and deploy automated coordinated attacks makes them particularly destructive for distributed systems such as IoT and Smart cities. Attacks such as RapperBot (August 2022), a recent variation of Mirai malware [17], infected a large number of IoT devices with a combined attack from over 3500 unique IPs, underpinning the importance of protecting resource-constraint devices from cyberattacks. Obfuscated memory malware (OMM) is a particularly notorious form of malware that employs obfuscation techniques to obscure its presence in the device memory and hide its activities such as code scrambling, command-and-control, string compression and encryption, and code injection to evade detection. Their polymorphic ability to change their behavior with each iteration also makes their goals impossible to understand, thus making it difficult to extract information about the intent of the malware. OMMs include some of the most dangerous viruses, such as Ransomware, Spyware and Trojans [18]. Thus, OMMs have certainly become powerful tools for infiltrating secure networks and stealing or destroying valuable information. Consequently, there has been a growing interest in the development of robust and efficient detection mechanisms that can analyze and identify OMMs in memory.

Recent advances in deep learning (DL) have led to an increased use of advanced neural networks algorithms, such as Convolutional Neural Networks (CNNs) and Long-Short Term Memory (LSTMs), to detect and identify malware [19]. However, it is important to consider the complexity and sporadic nature of OMMs activity patterns, especially when performing multiclass detection (i.e., identifying individual attack types), as the nature of these malware makes the task more challenging. While researchers have implemented a wide range of singular and hybrid models for malware detection, most of the research has focused on binary detection only, which detects the presence/absence of attack within a system. Multiclass detection is critical for embedded and constrained devices to optimize their security strategy by observing and investigating the specific nature of each attack type and devising more custom malware prevention systems. Another major challenge is that most existing methods for detecting OMMs fail to deliver sufficient detection accuracy while maintaining small model sizes. Very few works have focused on models specifically tailored to resource-constrained environments, as most DL methods consist of many layers and a large number of parameters, resulting in model sizes that exceed 1MB and are impractical for deployment on end devices [15].

Compared to LSTM which has a tendency to forget patterns after a while, Bi-directional LSTMs, have the unique ability to extract rich feature representations and model complex patterns while considering both past and future contexts [20]. They are adept at retaining or discarding critical and redundant information through the use of gates, thus reducing the resources required. These capabilities are useful to overcome the challenges posed by OMMs in targeted systems. Our model combines CNN and Bi-LSTMs because CNNs excel at mapping features, thereby eliminating the need for feature selection. They also reduce the number of learnable parameters by sharing weights and sub-sample data through pooling layers, reducing the dimensionality of data and computation requirements. Therefore, employing CNNs and Bi-LSTMs as feature extractors and classifiers, respectively, in resource-constrained environments enables us to develop robust and resilient memory attack detection techniques to counter the advanced tactics employed by OMMs.

Overall in this work, we address the associated issues and propose a DL-based OMM detection and attack classification system suited for resource-constrained systems, and make the following contributions:

- We designed a hybrid CNN-BiLSTM architecture for the detection and classification of OMM types (multiclass detection). Our model implements a two-layer CNN block, followed by a two-layer Bi-LSTM block to extract high-dimensional feature representations and capture the sequential correlations, respectively.
- Through extensive tuning of model parameters, we constructed two distinct models, namely CompactCBL and RobustCBL. While they vary slightly in performance, both models are embeddable in resource-limited IoT devices.
- Extensive evaluation on the most recent OMM dataset (CIC-Malmem-2022) demonstrates our models’ superior performance to other competing models and provides a tradeoff between performance and resource.

Acronyms and notations used in this paper are specified in Abbreviations.

The rest of the paper is organized as follows. Section 2 provides an overview of the current literature on binary and multiclass obfuscated malware detection. Section 3 presents an in-depth look at our proposed method, while Section 4 details the performance results obtained from evaluating the CNN-BiLSTM approach on CIC-Malmem-2022 Dataset. Finally, Section 5 summarizes our study and outlines future perspectives.

## 2. Related Works

The accurate and efficient detection and identification of malware using machine learning is a subject of constant research, mostly due to the adaptive nature of some attacks and the constant emergence of new threats. Obfuscated malware poses a significant challenge to researchers as it is designed to evade detection by traditional security methods. Compounded by the fact that small IoT devices also need protection from these attacks, recent studies have focused on developing novel solutions to prevent obfuscated malware and adapting neural networks for devices with limited resources. On detecting obfuscated malware using machine learning and neural networks, the approaches taken in literature can be broadly categorized into two:

1. Detecting the existence of an attack, i.e., binary classification and
2. Classifying attacks into individual families or types i.e., multiclass classification.

The following sub-sections present the approaches taken by prior studies.

### 2.1. Detecting the Presence of an OMM Attack (Binary Classification)

Lee et al. [21], present an anti-obfuscation classification method for Android malicious applications that integrates Recurrent Neural Network (RNN) and CNN for their ability to learn patterns in sequence data. VirusTotal was used to collect data for this study, extracting application package name, authentication data, permission, and intention features from multiple short strings. The authors attempted to achieve both anti-obfuscation capability and a lightweight design by focusing on package and certificate data as inputs because they are simple but easy to interpret for validation. The evaluation steps included five separate models, from Ngram feature model to using CNN and RNN. A model that combines CNN and RNN with additional features about permission and taken actions attains the highest FPR (False Positive Rate) detection rate of 97.7% for binary classification. The evaluation dataset is also particularly not made of obfuscated malware, but rather random Android attack samples. Therefore, the reported results are unlikely to hold true when tested on obfuscated dataset. Furthermore, the size of the model weights is about 4 MB, and the training time per epoch reaches 8 min, which is infeasible for IoT devices.

In [22], authors examine the challenge of making traditional detection systems resistant to obfuscation in malware. The authors analyze various popular virus scanners and conclude that current detection algorithms are significantly less effective when faced with obfuscated malware. The objective of the study is to develop a model called Obfusifier, which while trained on unobfuscated data, can accurately predict when it encounters obfuscated malware. For that, the authors assume certain parts of malware code, such as API invocations and control messages, cannot be obfuscated by attackers without breaking the functionality of the malware code. This hypothesis is utilized to extract features centered on API usage from the VirusShare malware repository. The proposed model employs JITANA [23] to generate method graphs for understanding call relationships in VirusShare apps. The model utilizes Google-provided API lists to only retain unchangeable, simple android API graphs. Using depth-first search, the model analyzes critical API call frequencies to gain insight into the generation of sensitive communication paths. Eight features, each from the original graph, simplified graphs and sensitive paths, are used to build the dataset. Decision Tree, Random Forest and Support Vector Machine are applied to the data with 10-fold cross-validation. After extensive training, the highest accuracy of 95% is achieved using Random Forest for binary classification. While the model demonstrates detection ability to some degree of obfuscation, it is unable to detect malicious inter-app communications between obfuscated attackers and is unable to detect malware if obfuscation is performed on the native code instead of applying at runtime.

Baek et al. [24] addressed the challenge of obfuscated attacks on IoT devices in smart cities by proposing a two-layer detection model called 2-MaD. The study explores the potential of deep learning to enhance detection capability against obfuscation. The preprocessing involves performing a static analysis on opcode sequences extracted from ELF files from the KISA-datachallenge2019-Malwares.04 dataset. The main operation of the model concentrates on real-time analysis of API functions and process memory, which are subjected to tensorization, selection, and classification using the EfficientNet-B3 model CNN technique. The performance of the study is hindered by the loss of features due to the fixed feature size for IoT environments and their resource-constrained nature. The authors construct the model with a large number of cells in hidden layers, resulting in a substantial number of parameters in the second layer of the first stage, which may pose issues while implementing on said systems.

Stacked Ensemble was employed in [18], to classify memory malware. They design and create a dataset comprising very recent and advanced obfuscated attacks on device memory, where features were extracted from memory dumps using VolMemLyzer, a feature extractor for machine learning systems. The dataset consists of 58,596 samples, containing 15 attack types belonging to Ransomware, spyware and trojan families. At first, they evaluate the detection accuracy of the dataset through traditional algorithms such as Random Forest, Decision Tree, kNN, SVM and Linear Perceptron. Of those methods, Random Forest attains the highest accuracy of 98%. To further increase the accuracy, an ensemble model using stacked generalization was created with Naïve-Bayes, Random Forest and Decision Tree as base and Logistic Regression as Meta-Learner. The method improves the accuracy to 99.02%. However, the study did not implement attack classification, which is crucial for dealing with the increasing number of attack types and variations. Additionally, the model takes 1 s to predict only 125 samples, which can create latency in networks that require constant data streaming and monitoring, such as IoT systems, making it not efficient for real-time monitoring scenarios.

The CIC-Malmem-2022 dataset has garnered considerable attention in recent literature. In [25], the authors employ oversampling and XGBoost as preprocessing techniques to combat class imbalance in the dataset, and after evaluating multiple algorithms, their experiments saw that Random Forest and Multilayer Perceptron(MLP) surpass the performance of other models, achieving an accuracy rate of almost 100%, although simply for detection. Although the study is commendable, it lacks the evaluation of individual attack classification. Furthermore, the ratio of attack and benign samples is equal in the dataset (Section 3), thus raising questions about the necessity for SMOTE, which is typically used for imbalanced datasets. Another contribution is the study by Louk et al. [26], who specifically analyze the performance of tree-based ensembles for binary classification of malware, with a specific focus on evaluating the performance of Xgboost, Catboost, LightGBM, and Random Forest. However, the authors do not specifically concentrate on building a model for obfuscated malware and instead aim to assess the generalizability of the algorithms across various contexts. The results of the study highlight Xgboost as the top performer.

The authors conducted a comprehensive evaluation of various baseline algorithms for binary classification of the dataset in [27]. The study aimed to assess a wide range of machine learning and deep learning models, such as Decision Tree, Gradient Boosting Machine, Logistic Regression, Multi-Layer Perceptron and Long-Short Term Memory Networks. The results showed that among the evaluated models, Logistic Regression stood out as the best performer, with an accuracy of 99.97% and an AUC score of 1.0. However, again no multiclass investigation was carried out.

Nonetheless, it is apparent that there is a limitation in the literature in regard to the classification of individual attacks or attack families, which would be an important aspect to consider.

### 2.2. Detecting Attack Families or Attack Types (Multiclass Classification)

Kim et al. [28] delve into the use of both global and local features for detecting obfuscated malware, with a specific focus on the efficacy of a variational autoencoder in defining a latent space to learn complex distributions of virus 2D vectors, which were transformed into grayscale images to gain insight into global features. The research also meticulously examines the capabilities of local features in describing distinct variations in small patterns within images. The team leveraged a Generative Adversarial Network model to extract global features, as it enables the production of data through random sampling, thereby expanding the range of detectable malware. The proposed method was rigorously evaluated using the Microsoft BIG 2015 dataset, where samples were used for image generation. The autoencoder was trained with a different mixture of Gaussian distributions, then transfer learning was used to build the detector, where the encoder is reused to inherit the original capacity of learning. The model achieves a detection accuracy of 97.47% for the detection of eight classes of malware. However, the study did not evaluate the optimal selection of features, which could have reduced redundancy in the feature space, resulting in improved performance, and neither did they consider any constraints in terms of resources. The assumption that the file sizes of the graphs based on the feature are sufficient to deduce whether an app contains malicious content or not is not reliable in cases where the attacker employs compression attributes.

A hybrid deep learning model was introduced in [29] that incorporates a deep belief network with a gate recurrent unit (GRU) for the detection of obfuscated malware on Android devices. They aim to make their model less error-prone by adding dynamic features collected at runtime alongside normal static features. The DBN feed-forward network is employed for static features, but its inability to detect dynamic obfuscated malware requires the addition of GRUs, due to their ability to capture temporal dependencies in the input data. Despite the authors’ effort to design a relatively simple model comprising only 5 to 25 neurons, the error rate remains substantial, ranging from 8 to 35%. The study does not prioritize the establishment of an optimal balance between model complexity and accuracy, but instead, it seeks to demonstrate that an increase in the number of neurons can aid in the more accurate detection of disguised malware. The amount of data used in the study is relatively small, only 280 samples, and although they show that an increase in neurons per layer can improve detection accuracy, the false alarm rate is still considerably high. Further evaluation with more samples might be of use to improve the overall performance of the model.

In [30], an approach to detect obfuscated malware was proposed, which uses the network signal behavioral signatures and API call patterns derived from a simulation environment established using a Cuckoo sandbox. More than 270,000 samples were generated of which 32000 were used. The authors reiterate the assumption made in [22] that API calls and behaviors cannot be obfuscated and uses those features to detect malware. To identify critical features, the authors utilized an Information Gain ratio, which was incorporated into a Random Forest algorithm to not only detect malware attacks but also classify them into five classes. By focusing on the sequences and frequency of API calls and augmenting this feature set with 55 additional features to enhance family classification, the system provides a granular understanding of malware behavior in the simulated environment. However, despite claims of reduced feature space and improved computational efficiency, the authors fail to provide empirical evidence to support these claims.

Mezina et al. [31] present a method to address the problem of obfuscated malware from memory information, utilizing the CIC-Malmem-2022 dataset created by [18], as a means of evaluating the efficacy of a dilated convolutional network (DCNN) in identifying hidden malware. DCNNs have been identified as a powerful tool for this task due to their ability to cover a large field of information, which is useful for uncovering hidden malware. By increasing the dilation area, the filter covers a larger feature space while covering the same parameter size. The study conducts a thorough evaluation of the proposed method through both binary and multiclass classification, achieving a high level of accuracy, specifically 99.92% and 83.53% respectively, with the latter being for classifying four major families of attacks in the dataset. The model comprises four layers, each containing two convolutional layers, with neurons ranging from 32 to 256. The size of the model poses an obstacle to implementation on small devices as it demands significant computational costs.

Based on the analysis of the prevailing literature in the domain, it is apparent that insufficient attention has been paid to building and evaluating detection models for recent obfuscated malware that are capable of functioning within limited system requirements. In light of this, our study tries to bridge this gap by proposing a robust and resource-efficient detection algorithm, the performance of which was rigorously evaluated against the most recent threats.

## 3. Proposed Methodology

In this section, we present the process of our fusion DL model. First, we summarize the limitations of current methods and architectures for the detection of OMM, particularly for attack type identification and resource constraints which motivated us to propose an innovative, robust and compact DL-based architecture. This is followed by an elaborated view of the proposed detection model architecture and its characteristics. Finally, we discuss the steps taken to ensure the model’s capability to operate in a resource-scarce environment.

### 3.1. Motivation for and Innovation of the Model

The increasing prevalence of obfuscation techniques and the constant emergence of new attacks targeting connected systems of all forms make it imperative to have robust attack detection and identification mechanisms. However, the literature on multiclass detection of obfuscated attacks is very limited, and among these works, models devised to operate in resource-constraint setting are almost non-existent. DL models possess the ability to scale their sizes, and with adequate tuning or pruning, a smaller model can be developed to identify complex attacks. Previous works have utilized the two primary algorithm types best suited for obfuscated malware detection, CNN and RNNs. Such works include combining CNNs with more generic RNNs [21] or CNN-LSTM hybrid models [32], etc. However, these models have not been trimmed to reduce the model size (lightweight models) so that they can be used in resource-constrained IoT devices, especially for specific attack detection.

However, traditional RNNs frequently encounter vanishing gradient problems [33] when applied to deceptive malware, which was solved by LSTMs using forget gates [34]. It was also observed that unidirectional LSTMs struggle to capture long-term dependencies in data streams with complex patterns, as the one-way analysis is prone to overlook hard-to-detect features. The improved version with superior performance is Bi-LSTM [35], whose ability of advanced pattern analysis makes it a good candidate to counter evasive attack techniques. The concurrent use of CNN and Bi-LSTM is elaborated further in the following section.

### 3.2. Proposed Architecture

**Figure 1:**
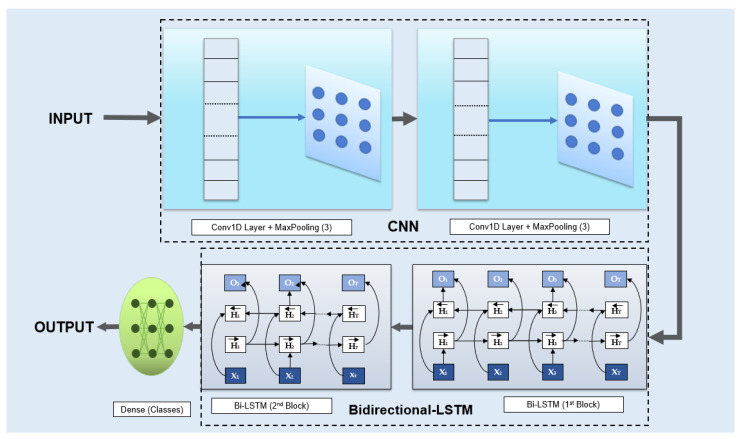
Proposed CNN-BiLSTM Model.

Prior research on DL algorithms addressing challenges mentioned in the previous section has been limited, with an insufficient evaluation of their ability to detect malware types in case of advanced obfuscation attacks hidden deep in device memory. Therefore, our study focuses on creating a model to distinguish these complex attacks while being deployable in embedded devices with limited memory and computational power. This approach of advanced OMM multiclass detection in resource-limited environments is, to the best of our knowledge, the first of its kind. We propose a hybrid stacked approach, consisting of CNN and Bi-LSTM, as both algorithms offer scalable architecture making the tradeoff between performance and model size possible. CNNs enable a deeper understanding of the input data, while Bi-LSTM is effective for analyzing the polymorphic behavior of obfuscated malware in network communication through pattern recollection. Our proposed architecture can be visualized in Figure 1.

CNNs play a crucial role in identifying obscured patterns by employing layers of interconnected neurons with respective weights and biases. The core components of CNN are the hidden layers, which modify the input data to attain the desired output representation. One or more of these hidden layers are convolutional, which leverage(s) filters to alter the input data or previous hidden layer weights and biases. Networks with more layers and neurons learn more intricate patterns but require additional resources, which is why we tuned the number of filters to attain a balanced trade-off. During training, filters are optimized through convolving [36] (Equation (1)) to detect pertinent features, which constitutes the principal operation of the model that prepares the outputs for the next layer.

Let *x* be the input to the CNN, **$w_{1}$** and ***w*** be the weights of the previous and present layer respectively, ***b*** be the bias terms and ***f*** be the activation function. Then, the output of a convolutional layer can be calculated as:

(1) $$w=f(w_{1}\times x)+b$$

where × represents the convolution operation.

To extract the most significant features while discarding less informative information, feature spaces between convolutional layers are subjected to down-sampling through spatial pooling operation [37]. For this purpose, MaxPooling with a pool size of 3 is utilized to reduce the spatial dimension. Due to the feature extraction ability of CNNs, we could discard any pre-processing or feature selection procedure, which could have otherwise consumed more memory resources. This feature greatly contributes to building a resource-limited model without compromising the malware detection accuracy. We use two CNN blocks to interpret data, within each is a Conv1D layer followed by a MaxPooling Layer. Conv1D is a type of CNN layer that is commonly used for sequence data, that applies 1D convolving, ie., a sliding window technique to the input. As the detection of malware often needs the analysis of sequences of byte code, Conv1D is an extremely useful approach to extract features from the data. The optimum CNN structure can be obtained through a fine-tuning process for a particular dataset. In our study, the CNN part of the architecture consists of the following components:

**Input → 1st CNN Block (Conv1D+MaxPool) → 2nd CNN Block (Conv1D+MaxPool) → To BiLSTM**

Note that this structure has been achieved considering the whole proposed CNN-BiLSTM model. Now, we describe the usage of Bi-LSTM for our purpose. Although the existing literature has illustrated the effectiveness of LSTMs used in conjunction with CNNs, we have observed their limitations in detecting obfuscated malware. We use Bidirectional LSTM because it is an improvement over LSTM which operates by processing input sequences in both forward and backward directions, incorporating hidden states from both directions. Thus it allows the network to consider the input sequence in both previous and present contexts [38]. Hence, this bidirectional approach is essential in addressing the deceptive nature of hidden malware. The output of the CNN block is propagated to the Bi-LSTM section at first, and then at each time step, forward and backward LSTMs process the series of data in opposite directions, producing two hidden states. States for each step are then concatenated to make predictions about the present sequence, or the present sample. Due to this double operation, Bi-LSTMs outperform unidirectional LSTMs, which was also observed in our evaluation (details in the next section).

In a similar way to selecting the CNN structure, our Bi-LSTM structure is also selected based on the same dataset and is comprised of two blocks, allowing secondary learning for the model to be performed on the sequences identified by the first block. The output from the second Bi-LSTM block is directed into a subsequent fully connected (dense) layer, comprising neurons corresponding to the total number of classes in the dataset. The resulting ’Class Output’ from this dense layer signifies the predicted classification for each instance, categorizing it as either ’benign’ or an attack category. This class output value indicates the probability of a sample belonging to a particular benign/attack category based on the model’s learned patterns. As such, the Bi-LSTM section of our proposed architecture looks like:

**CNN Output → 1st Bi-LSTM Block → 2nd Bi-LSTM Block → Dense Layer → Class Output**

Within our proposed method, we experimented further to evaluate the impacts of size and parameters, with the aim of achieving the highest possible detection accuracy while keeping the model size as small as possible, never exceeding 1 MB. This is crucial for real-time applications and embedded systems, as many smart city applications require the use of IoT devices that need to run a DL model to produce responses in real time or quasi-real time. As shown in Section 2, if the model size is kept within 1 MB, most devices are capable of running the models in real time.

To evaluate the trade-off between size and performance, we created two models that follow the same architecture but differ in the number of parameters. The first model, named CompactCBL (Compact CNN-BiLSTM), was designed for extremely constrained devices and was kept under 600 KB for every classification task in our study. We then designed a second model, RobustCBL (Robust CNN-BiLSTM), which has a slightly larger number of parameters but still remains under the 1 MB threshold. The model size for this version is around 970 KB, with minor variations for each classification task. Our goal was to evaluate the tradeoff between the two so that they can be used on a wide range of resource-constrained devices, without compromising on accuracy or performance. The specific parameters chosen for both models are described in Table 1.

## 4. Experimental Results

To evaluate the effectiveness of our models in detecting obfuscated malware, we utilized the most recent and comprehensive dataset, CIC-Malmem-2022. This section provides a detailed description of the dataset, along with a performance analysis of our models. To ensure a thorough assessment of their capabilities, we conducted three distinct detection tasks: (i) binary attack detection, (ii) attack family detection and (iii) identification of individual attack types. Moreover, we compared our approach with the existing literature to assess the validity of our approach.

The experiments were conducted on an HP EliteOne Desktop, equipped with a 64-bit Windows 10 Education operating system and an Intel(R) Core(TM) i5-7500 CPU @ 3.40 GHz 3.41 GHz processor, with 8 GB RAM. For model building and testing, we utilized popular libraries such as Pandas, Numpy, TensorFlow, Keras and Sklearn.

An 80–20% division of the dataset was performed to generate training and testing sets. As such, the train set contained 46876 samples while the test set had 11,720 samples. Both sets were stratified to ensure all classes exist in both sets. The models were then constructed using the parameters described in Table 1. During the training phase, batch size of 64 was used to avoid any additional latency, which was also done for the test set to ensure a consistent evaluation process. After the training phase, the model’s weights were saved and loaded for evaluation on the test set. We opted to use the Adam optimizer to update the weights while tracking the loss with the categorical cross-entropy function, which is known for its ability to handle multi-class problems.

### 4.1. Dataset

The CIC-Malmem-2022 dataset [18] was created by the Canadian Institute of Cybersecurity and made publicly accessible in 2022. The dataset was created with a focus towards memory-based obfuscated attacks, containing malware from very recent real-life cyberattacks. The samples were created from malicious memory dumps created by using VolMemLyzer [39], a technique for extracting significant feature representations from real-time network communications. Malmem-2022 contains 58,596 samples in total, with 56 features in each sample. Exactly half of the collection consists of benign network data, and the other half is made up of a variety of contemporary and old obfuscation-based cyberattacks. These attacks can be broadly classified into three families, namely Trojan, Spyware and Ransomware, which collectively comprise 15 distinct attack types. The diversity presented by these attacks provides a unique opportunity to evaluate detection models that can perform against an array of threats, where the attacks range from the browser hijacker CWS malware (2003) to the ever-changing Trojan Scar whose patterns are changing even after 10 years since first detection, to the Ransomware Conti that surfaced in the pandemic era of 2020. This dataset was selected for our study because evaluating new models on old datasets does not translate to efficacy in the real world, as the performance of models heavily depends on the features associated with the attacks and their complexity [40].

**Figure 2:**
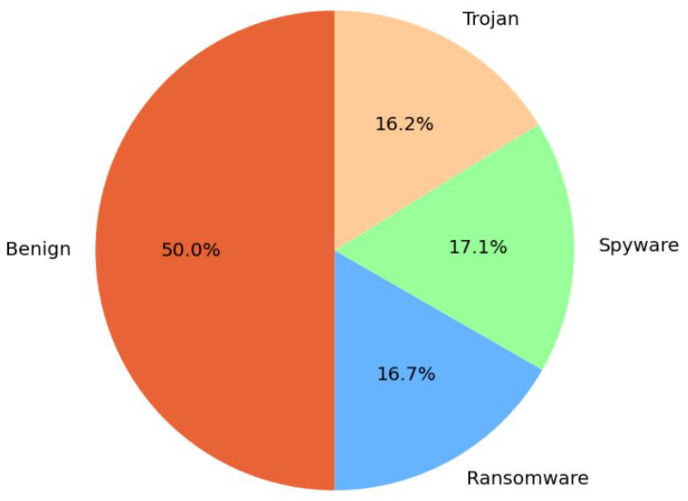
Distribution of Attack Families in CIC-Malmem-2022 Dataset.

Figure 2 shows the class distribution between benign and attack family categories in CIC-Malmem-2022, which comprises 50% Benign Samples, 16.7% of Obfuscated Ransomware, 17.1% Spyware and 16.2% Trojan malware.

We then illustrate the breakdown of individual attacks in Table 2. The sample number of all attacks is almost the same, except Transponder, Gator and Shade which contain over 2000 samples. TIBS has the lowest number of samples for any class.

### 4.2. Model Evaluation Metrics

To evaluate models’ performance in detecting OMMs and its validity for application in resource-constrained devices (IoT devices) used in smart city applications, we measured several widely used metrics that are used to determine the detection accuracy of a machine learning model. At first, we define the following:

- True Positive (*TP*) = number of samples the model correctly detects as attack.
- True Negative (*TN*) = number of samples the model correctly detects as benign.
- False Positive (*FP*) = number of samples the model incorrectly detects as attack.
- False Negative (*FN*) = number of samples the model incorrectly detects as benign.

Accuracy (*ACC*): The proportion of correct detections (True Positives and True Negatives) made by the model to the total number of detections (*N*):

(2) $$ACC=\frac{TP+TN}{N}\times 100$$

Precision (*P*): The specified percentage of positive predictions which are accurate:

(3) $$P=\frac{TP}{TP+FP}\times 100$$

Recall (*R*): Proportion of actual benign classes that the model is able to correctly identify:

(4) $$R=\frac{TP}{TP+FN}\times 100$$

F1-Score (*F*1): The harmonic mean of precision and recall:

(5) $$F1=\frac{2\times R\times P}{R+P}$$

To determine the feasibility of integrating the proposed model into resource-constrained devices, two further metrics, (i) model weight size and (ii) detection speed, were calculated and compared with existing works. In the following sections, we demonstrate the results obtained from our experiments to determine our models’ performances in binary, attack family and individual attack detection.

### 4.3. Binary Attack Detection

At first, we experimented with binary attack detection using our proposed models and contrasted their findings with the existing literature. Our RobustCBL and CompactCBL models both demonstrate similar performance, attaining an accuracy rate of 99.96% and 99.92%, respectively. Notably, both models achieved almost perfect scores (1.0) on additional evaluation metrics such as Precision, Recall and F1-score. However, given the relatively straightforward differentiation between two distinct feature types of classes, wherein one is markedly different from the other, it is typical for models to achieve near-perfect accuracy, as evidenced in Table 3, where we compare our model results with existing literature. We observe that our model performs at perm or even better than the other models. Further assessments are required to fully assess the models’ potential for attack family and individual attack detection.

In alignment with the studies in [18,31], our models have been evaluated with an 80–20% division of training and test data (as we mentioned before). However, the LSTM model in [27] employed a 70–30% split. To ensure a fair comparison with LSTM model, we additionally conducted another experiment with the same split. Results with this split show that RobustCBL and CompactCBL attain accuracies of 0.9994 and 0.9985, respectively, compared with 0.9943 obtained by the LSTM model in [27].

### 4.4. Attack Family Detection

Although binary detection results are promising, merely detecting the existence of an attack is not enough. To effectively prevent attacks and secure a network through robust security policies, it is crucial to know the attack types and their nature. In this section, we built models to identify three broad attack families and a benign category, i.e., four classes in total. Table 4 presents the results of our family detection performance compared to the existing literature.

The two proposed models attain 84.56% (RobustCBL) and 84.22% (CompactCBL) detection accuracy and outperform other existing works. Note that RobustCBL achieved slightly higher accuracy due to its larger parameter size, whereas CompactCBL performed remarkably well despite being nearly 40% smaller than RobustCBL. Moreover, both models outperformed Mezina et al.’s Dilated CNN model [31] by 1.03% and 0.7%, respectively, which is the sole other work to evaluate multiclass classification on this dataset and reported by the authors. They also evaluated the performance of Decision Tree, but with less successful outcomes.

Considering other metrics, our models attain 0.85 and 0.84 precision, 0.85 and 0.84 recall and 0.84 F1-score for both cases against 0.76, 0.75 and 0.75, respectively, by DCNN, making significant improvements over existing works. These metrics hold particular relevance in the context of malware detection. The classwise performance comparison in detail are presented in Table 5. Results from both models reveal that the classifier performed remarkably well in identifying benign samples, whereas obfuscated attack samples posed a greater challenge. Nevertheless, our models perform better than existing works in this regard in most cases. The difficulty in detecting obfuscated attack samples can be attributed to the absence of obfuscation in benign samples, which makes them comparatively easier to detect. Additionally, ransomware attack detection is more challenging than that for other attacks. This can be attributed to the fact that most Ransomware attacks in the dataset have originated quite recently, and uses advanced obfuscation technologies to hide any characteristics that would help categorize it as malware.

Size constraints represent another key factor in assessing tradeoffs between size and performance. Thus, we compared our models’ weight size to that of DCNN. However, as no model size for DCNN was reported in [31], therefore, we reconstructed an approximate model based on their description, which yielded a size closer to 6 MB, rendering it unsuitable for most sensors and IoT end devices. As alluded to in Section 3, resource-constrained devices, such as IoT devices for real-time or quasi-real-time applications in smart cities, where response times are within 1 ms, require ML models of at most 1 MB size. Therefore, our architecture, particularly the CompactCBL model, demonstrated the ability to attain greater performance than DCNN [31] while being significantly smaller, only 577 kB, or 1/20th the size of DCNN.

### 4.5. Attack Type Detection

It is important to note that individual attacks in an attack family can possess unique characteristics and different obfuscation techniques that require customized detection and mitigation strategies. By being able to distinguish among different variants, the effectiveness of security measures can be enhanced. For instance, the Trojan family consists of Zeus, which originated in 2007, and Scar attacks, the latest version of which appeared in 2019 [41]. As such, we can assume there would be significant differences between the mechanisms of Zeus and Scar, as well as the associated patterns or features.

Therefore, evaluating our model’s performance in detecting all 16 individual classes separately would provide insights into its ability to detect individual attack types.

To the best of our knowledge, our study is the first one that attempts to identify all 15 separate attack types in the dataset. We show the evaluated results of RobustCBL and CompactCBL in Table 6, where they attain detection accuracy of 72.6% and 71.42%, respectively. As no reported works exist in the literature on attack type detection on the Malmem-2022 dataset, these results could not be compared.

As we can see, RobustCBL performs better than CompactCBL in all four metrics. An analysis of the model’s predicted classes shows that the benign class exhibits very high accuracy, as it contains features that are not obfuscated and hence easy to distinguish from attack class features. As expected, since there are many classes, identifying each class with high accuracy is difficult because extracting features with sufficient enough distinguishable characteristics among a large number of classes are challenging. Our observations show that some of the ransomware attacks (Ako, Shade, Ransomware Pysa, Maze or Conti) are misclassified as one of the Trojans, specifically, Zeus attack. Within the Spyware family, we observe that some of the 180 solutions and CWS samples are misclassified as Transponder attacks. In general, detecting Ransomware type of attacks was more difficult than Spyware or Trojan type of attacks.

### 4.6. Detection Speed

Security measures placed in a network should be able to make prompt and decisive actions in response to incoming attacks. This is more critical in IoT systems where real-time detection is essential. As alluded to before, sensors and end devices in IoT systems should have the ability to detect signals within a time frame of less than 1 ms. This motivated us to compare the speed of our proposed models with existing works [18,31], shown in Table 7. Results demonstrated CompactCBL’s superior performance, classifying each sample in only 0.255 ms, aided by its compact model size without significant accuracy compromise, as previously detailed. RobustCBL detected sample at a speed of 0.384 ms, indicating both models’ suitability for IoT devices. While DCNN’s detection speed is within the acceptable time limit, its model size makes it unsuitable for many IoT devices. On the other hand, the stacked ensemble-based model is extremely slow for real-time attack detection.

### 4.7. Applicability of the Proposed Models

Our CNN-BiLSTM-based models, CompactCBL and RobustCBL, embody the necessary properties for integration into IoT-fuelled Smart City applications. As urban spaces increasingly adopt sensor-driven systems, sectors such as guided parking in heavily populated areas [42], detecting violence through video surveillance and real-time monitoring of communication infrastructures (such as railroads and highways) are key areas that use sensors to monitor and relay the information to the processing layer. IoT sensors used in these applications are vulnerable to cyber threats including obfuscated memory malware attacks. In the event of an attack, the compromised sensors can affect the operational conditions of a smart city including traffic conditions, urban environment and law enforcement. Likewise, monitoring systems assessing water, air and noise quality, which are foundational for urban sustainability, demand efficient memory malware detection frameworks. As mentioned in Section 1, OMMs are harder to detect than regular malware, and as such, our models aim to secure the constrained devices used in these applications, bolstering their resilience against complex cyber threats.

Validated on the most recent OMM dataset CIC-Malmem-2022, our models outperform relevant existing models, achieving high accuracy for binary and attack family (4-class) identification, with good accuracy for individual attack identification (16-class). Most notably, CompactCBL, despite being the smallest, outperforms every other algorithm except RobustCBL. The model achieves 99.92%, 84.22% and 71.42% for binary, family and individual attack detection, while RobustCBL attains 99.98%, 84.56% and 72.60% accuracy, respectively. The larger model size aids RobustCBL in producing higher accuracy, still, its size of around 970 KB is easily implementable for constrained devices. The small size of CompactCBL makes it faster with a detection speed of 0.255 ms/sample, making it more suitable for implementation in various sectors mentioned above. As such, for broader applicability, the two models introduced by this study can be used in defense of a wide range of IoT and small-scaled embedded devices used in smart cities, where real-time response is required.

## 5. Conclusions and Future Works

Sophisticated evasive mechanisms against detection have made OMMs harder to detect than other types of malware, and as such their usage for malicious purposes has skyrocketed in recent years. Though large DL models with huge parameters exist to prevent these attacks, they cannot be used in small-scale systems such as IoT networks in smart cities and other applications. In this paper, we present a robust system for OMM detection in resource-constrained devices. We utilize CNNs for their ability to extract obscure features from malware memory, and Bidirectional LSTMs for longer and context-aware pattern analysis. To evaluate the effectiveness of our method, we built two models, (i) CompactCBL and (ii) RobustCBL, and evaluated their performance using the recent OMM dataset. Our models outperformed existing models in terms of widely used detection performance metrics and the time required for detection. Additionally, this study helps to gain insights into obfuscated ransomware’s enhanced deception ability compared to other attacks.

While our proposed models demonstrate advancement in obfuscated malware detection, there are scopes for further improvements. Specifically, enhancing accuracy in detecting granular multiclass (individual) attack types remains a challenge. Thus, our future efforts will focus on addressing this limitation. This will involve devising innovative architectures to enhance the identification of individual attack types, as well as unknown or zero-day attacks, for which we will investigate semi-supervised/unsupervised learning models into our framework while keeping the model size implementable in sensors. Furthermore, future studies will also target evaluating the models on a real-world IoT-based smart city application (Guided Parking) under various obfuscated malware attacks.

## Figures and Tables

**Table 1:** Specification of CompactCBL and RobustCBL designed for our evaluation.

Model	Layers	Parameter
CompactCBL	Conv1D_1 Layer	32×1
	Kernel Size	3×1
	MaxPooling	3×3
	Conv1D_2 Layer	64×1
	Kernel Size	3×1
	MaxPooling	3×3
	Bi-LSTM_1	32 Neurons
	Bi-LSTM_2	16 Neurons
	Dense Layer	Number of Classes
RobustCBL	Conv1D_1	48×1
	Kernel Size	3×1
	MaxPooling	3×3
	Conv1D_2	64×1
	Kernel Size	3×1
	MaxPooling	3×3
	Bi-LSTM_1	42 Neurons
	Bi-LSTM_2	32 Neurons
	Dense Layer	Number of Classes

**Table 2:** Distribution of attack samples in Malmem dataset.

Class	Type	Sample Number	Proportion (%)
Benign	-	29,298	50.00
Ransomware	Ako	2000	3.41
	Shade	2128	3.63
	Pysa	1717	2.93
	Maze	1958	3.34
	Conti	1988	3.39
	**Total**	**9791**	**16.7**
Spyware	180solutions	2000	3.41
	CWS	2000	3.41
	Gator	2200	3.75
	TIBS	1410	2.41
	Transponder	2410	4.12
	**Total**	**10,020**	**17.1**
Trojan	Reconyc	1570	2.68
	Refroso	2000	3.41
	Scar	2000	3.41
	Emotet	1967	3.36
	Zeus	1950	3.33
	**Total**	**9487**	**16.2**

**Table 3:** Binary attack detection performance in comparison with existing models presented in [18,27,31]. Note that performance metrics of existing models are values reported in respective studies.

Model	Accuracy	Precision	Recall	F1-Score	Size (KB)
RobustCBL	0.9996	1.00	1.00	1.00	967
CompactCBL	0.9992	1.00	1.00	1.00	575
DCNN [31]	0.9992	0.99	0.99	0.99	6037
LSTM [27]	0.9943	0.99	1.00	0.99	627
Decision Tree [31]	0.9990	0.99	1.00	0.99	Not given
Stacking [18]	0.9902	0.99	0.99	0.99	Not given

**Table 4:** Attack family detection performance in comparison with existing models. The performance metrics of comparing study are the values reported in [31].

Model	Accuracy	Precision	Recall	F1-Score	Size (KB)
RobustCBL	0.8456	0.85	0.85	0.84	971
CompactCBL	0.8422	0.84	0.84	0.84	577
DCNN [31]	0.8353	0.76	0.75	0.75	6041
Decision Tree [31]	0.7916	0.69	0.69	0.69	Not given

**Table 5:** Family-wise detection performance metrics of proposed and existing method. The performance metrics of comparing study are the values reported in [31].

	RobustCBL			CompactCBL			Dilated CNN [31]		
**Class**	**Precision**	**Recall**	**F1**	**Precision**	**Recall**	**F1**	**Precision**	**Recall**	**F1**
Benign	1.00	1.00	1.00	1.00	1.00	1.00	1.00	1.00	1.00
Ransomware	0.67	0.62	0.64	0.67	0.60	0.63	0.62	0.66	0.64
Spyware	0.69	0.77	0.73	0.70	0.72	0.72	0.67	0.76	0.71
Trojan	0.71	0.67	0.70	0.68	0.74	0.71	0.73	0.57	0.64

**Table 6:** Individual attack detection performance metrics of proposed method.

Model	Accuracy	Precision	Recall	F1-Score
RobustCBL (975 kb)	0.7260	0.73	0.73	0.72
CompactCBL (581 kb)	0.7142	0.72	0.71	0.71

**Table 7:** Detection speed of our models in comparison with existing models. The speed of [18] is directly reported in the study, whereas the performance speed of [31] is deduced from the reconstruction outlined in Section 4.4.

Model	Speed (ms/sample)
CompactCBL	0.255
RobustCBL	0.384
DCNN [31]	0.738
Stacked Ensemble [18]	12.50

## Data Availability

Not applicable.

## Abbreviations

The following acronyms and notations are used in this manuscript:

OMM	Obfuscated Memory Malware
IoT	Internet of Things
DL	Deep Learning
CNN	Convolutional Neural Network
LSTM	Long-Short Term Memory
Bi-LSTM	Bi-directional Long-Short Term Memory
RNN	Recurrent Neural Network
API	Application Programming Interface
FPR	False Positive Rate
RobustCBL	Robust CNN-BiLSTM Model
CompactCBL	Compact CNN-BiLSTM Model
MLP	Multi-layer Perceptron
GRU	Gate Recurrent Unit
DCNN	Dilated Convolutional Neural Network
*w*, $w_{1}$	Weights of convolution layers
*b*	Bias Terms
*f*	Activation Function
*x*	Input to convolution layer
